# Electron Transfer Flavoprotein Subunit Beta Is a Candidate Endothelial Cell Autoantigen in Behçet’s Disease

**DOI:** 10.1371/journal.pone.0124760

**Published:** 2015-04-27

**Authors:** Peng Chen, Weikang Yang, Yaping Tian, Shutao Sun, Guangyu Chen, ChunYan Zhang, Fuxin Ma, Yiping Xun, Lili Shi, Chunhe Yang, Lanqing Zhao, Yabin Zhou, Hongwu Du

**Affiliations:** 1 School of Chemistry and Biological Engineering, University of Science and Technology Beijing, Beijing, 100083, China; 2 Department of Clinical Biochemistry, Chinese PLA General Hospital, Beijing, 100853, China; 3 Core Facility, Institute of Microbiology, Chinese Academy of Sciences, Beijing, China; 4 ImmunoHunt Corporation, 139 Fengtai Rd, Beijing, 100071, China; Baylor College of Medicine, UNITED STATES

## Abstract

Behçet’s disease (BD) is a chronic inflammatory disease with multisystem involvement, and it is listed as a rare disease in the United States but is common in the Middle East, China, and Japan. The aim of this study was to identify novel autoantigens in Chinese patients with BD. First, the candidate autoantigens were screened by Western blotting, and the sequences of putative antigens were identified by LC-MALDI-TOF/TOF mass spectrometry. Next, the screened protein was cloned, expressed and purified. Then, an optimized ELISA was developed, and the serological criteria were evaluated using a large number of confirmed patients. One antigen with a molecular weight of approximately 28 kDa was identified as electron transfer flavoprotein subunit beta (ETFB). Positive reactivity was detected in recombinant human ETFB sera from 38 of 92 BD patients (41 %) and 1 of 90 healthy controls (1 %).

## Introduction

Behçet’s disease (BD) is a multisystem chronic vasculitis that was first described in the 5th century BC [1]. BD occurs most frequently in an area that coincides with the Old Silk Route, an ancient commercial route that stretched between the Mediterranean and the Far East [2, 3]. The pathogenesis of BD remains uncertain, and its diagnosis is still mainly based on the clinical syndrome [4]. BD is usually characterized by vascular injury and the triple-symptom complex of recurrent oral ulcerations, genital ulcerations and iritis [5, 6], and many organs, including the skin and the gastrointestinal organs, are typically involved in this disease [4].

Anti-endothelial cell antibodies (AECAs) were suggested to be involved in the autoimmune process of BD. AECAs bind to endothelial cell antigens and can be directed against endothelial cells in clinically relevant organs. Their effects on endothelial cells are thought to be associated with the vascular injury and damage that occurs in BD patients and have also been confirmed to be associated with autoimmune symptoms [7, 8].

Similar to many traditional autoimmune diseases, such as rheumatoid arthritis (RA) and Sjogren’s syndrome (SS), the various signs and symptoms of BD suggest the co-existence of a large number of autoantigens [9– 11]. Recently, heat shock protein 27 and prohibitin were successfully identified in our lab [12, 13]. However, many questions remain, especially the pathogenesis of BD is still unknown, and more AECA autoantigen/autoantibody pairs should be identified in BD. Therefore, the aim of this study was to further identify new AECA autoantigens in human umbilical vein endothelial cells (HUVECs) [14].

## Materials and Methods

### Subjects

Serological criteria were evaluated through the assessment of 364 samples in total. This study included 92 BD patients with an average age of 39 years (range, 14 to 66 years; 38 females and 54 males) who fulfilled the criteria proposed by the International Study Group for BD [15], 92 rheumatoid arthritis (RA) patients (average age, 34 years; range, 15 to 62 years; 81 females and 11 males), 90 Sjogren’s syndrome (SS) patients (average age, 51 years; range, 19 to 70 years; 86 females and 4 males) and 90 healthy controls (HCs) (average age, 25 years; range, 21 to 33 years; 69 females and 21 males). Initially, samples from 5 BD patients were collected in July 2013. The other samples were collected from September 2012 to June 2014 for a large-scale test using the ELISA method. All of the patients involved in the study were treated at the Chinese People's Liberation Army General Hospital. This study was approved by the Ethics Committee of the Chinese People's Liberation Army General Hospital, and each patient involved in this study provided written informed consent. Furthermore, written informed consent on behalf of the minors involved in the study was obtained from their guardians. The samples were collected, dispensed, aliquoted and stored at -80°C for further testing.

### Cell culture and protein extracts

The HUVEC line was purchased from the American Type Culture Collection (ATCC, MD) and cultured in F-12K (HyClone, UT) containing 10% fetal bovine serum (HyClone, UT), 0.1 mg/mL heparin (HyClone, UT), and 0.05 mg/mL endothelial cell growth supplement (HyClone, UT). HUVECs were lysed in RIPA buffer (Beyotime, Jiangsu, China) with 1% complete protease inhibitor cocktail (Sigma, MO). The extracts were aliquoted and stored at -80°C until further use.

### Indirect immunofluorescence assay

HUVECs were applied to coverslips and subsequently fixed with 4% paraformaldehyde. Next, BD and HC sera were added to the slides, and the slides were incubated for 1 h at 37°C. After washing 3 times, the slides were then incubated with a FITC-conjugated goat-anti human IgG secondary antibody (ImmunoHunt, Beijing, China) diluted 1:500 for 1 h at 37°C. Finally, the slides were examined by fluorescence microscopy (AMG, Bothell, WA). For the indirect immunofluorescence assay, the total ratio of cell fluorescence was obtained using Image J software (NIH, MD), and the results were compared among 3 representative patients in each group.

### Western blotting

Western blotting was performed as described elsewhere [16] with slight modifications. The cell lysate was loaded into the wells of a 12% SDS-PAGE gel. The proteins were then transferred to polyvinylidene fluoride membranes (Merck Millipore, MA) and blocked with 5% nonfat milk in PBS at 4°C overnight. Next, the membranes were incubated with serum from the first batch of five BD patients (1:500 dilution) or sera from five HCs (1:500 dilution) at 4°C for 12 h. The filters were then extensively washed 3 times with 5% PBST to remove the unbound antibodies. Finally, the membranes were incubated with horseradish peroxidase-conjugated goat anti-human IgG (ImmunoHunt, Beijing, China) (1:10,000 dilution) for 1 h at 37°C. ECL detection was performed in accordance with the manufacturer’s instructions (Applygen, Beijing, China).

### Protein recovery and immunoprecipitation

Two different bands located at approximately 28 kDa were excised and recovered to ensure the retrieval of the reactive band. Briefly, the bands were cut out of the gel and placed in a 1.5-mL centrifuge tube with 0.5 mL of ultrapure water at room temperature for 5 min. The gel in the centrifuge tube was ground with 0.7 mL of extraction solution (50 mM Tris-HCl [pH 7.9], 0.1 mM EDTA, 150 mM NaCl, 0.1% SDS) at 4°C overnight. Following centrifugation (12000 rpm, 15 min), the extraction solution was transferred to a dialysis tube in PBS and maintained at 4°C overnight. After centrifugation (12,000 rpm, 15 min), the extraction solution was transferred into an ultrafiltration tube (Sartorius, Germany). Finally, the proteins were recovered by centrifugation at 14,400 rpm for 30 min. The total recovered proteins (10 μg) were incubated with sera (equal volumes from BD patients with a positive band after Western blotting selection) at 4°C overnight. Thereafter, protein A sepharose beads (Sigma, MO) that had been washed with PBS were added and mixed overnight at 4°C. The immunoprecipitates were washed three times in 500 μL of PBS. Then, the immunoprecipitates were suspended in sample loading buffer and resolved by SDS-PAGE.

### Mass spectrometry identification

The excised gel pieces were destained with a mixture of 25 mM NH_4_HCO_3_ and 50% acetonitrile and subsequently dried by vacuum centrifugation. Then, the gel pieces were reduced for 2 h in 10 mM dithiothreitol. After cooling to room temperature, the dithiothreitol solution was replaced with roughly the same volume of 25 mM NH_4_HCO_3_ containing 55 mM iodoacetamide and incubated for 45 min at room temperature in the dark. Then, the gel pieces were covered with 20 μL of 0.05 M NH_4_HCO_3_ buffer with trypsin (Sigma, MO). Digestion was performed overnight at 37°C. Finally, the recovered target proteins were identified using a 5800 Proteomics Analyzer. The mass spectrometric data were analyzed using a LC-MALDI-TOF/TOF mass spectrometer (Applied Biosystems, Foster City, CA) at the Institute of Microbiology of the Chinese Academy of Sciences, as described previously [17]. The mass spectrometric data were further analyzed using the Mascot bioinformatics database search engine (Matrix Sciences, London, UK; www.matrixscience.com).

### Protein expression and purification

The protein expression and purification procedure was performed according to our routine method [18]. Briefly, total RNA was isolated from HUVECs using TRIzol reagent (Invitrogen, CA). RT-PCR was performed according to the manufacturer’s instructions (Fermentas, MD). The human ETFB protein was overexpressed in *E*. *coli* BL21 cells, and the recombinant protein was purified using Ni-NTA resin (Qiagen, Hilden, Germany). The protein concentration was determined with the BCA assay kit (Biosynthesis Biotechnology, Beijing, China). Purified recombinant protein was confirmed by mass spectrometry (Applied Biosystems, Foster City, CA).

### ELISA

ELISA based on the human recombinant ETFB protein was performed as described previously [16]. Briefly, human recombinant ETFB protein (300 ng/mL) was used to coat a 96-well microplate (Corning, NY) overnight at 4°C. After three washes with PBST, each well was blocked in 200 μL of 5% goat serum for 2 h at 37°C. Then, the plate was incubated with 100 μL of sera (1:100 dilution) for 2 h at 37°C. After three washes, 100 μL of goat anti-human IgG HRP (ImmunoHunt, Beijing, China) (diluted 1:10,000) was added to each well, and the plate was then incubated for 1 h at 37°C. Next, the plate was washed three times, and 50 μL of tetramethylbenzidine (TMB) A (0.1 M citric acid, 0.2 M Na_2_HPO_4_, 0.6 g hydroperite/L) and 50 μL of TMB B (5 mM citric acid, 0.4 mM EDTA-Na_2_, 0.2 g TMB/L) were added to each well. Then, the plate was incubated in the dark at room temperature for 5 min. Finally, the reaction was stopped by adding 50 μL of 2 M H_2_SO_4_. The absorbance in each well was measured with a plate reader at 450/620 nm (Tecan, Hombrechtikon, Switzerland).

### Statistical analysis

The data and t-test were analyzed using SPSS software (Version 17, Chicago, IL). *P* values less than 0.05 were considered significant. The threshold for defining a positive result was a value higher than that of the healthy controls (mean + 3 SD).

## Results

### Endothelial cell autoantigens are expressed in HUVECs

**Fig 1:**
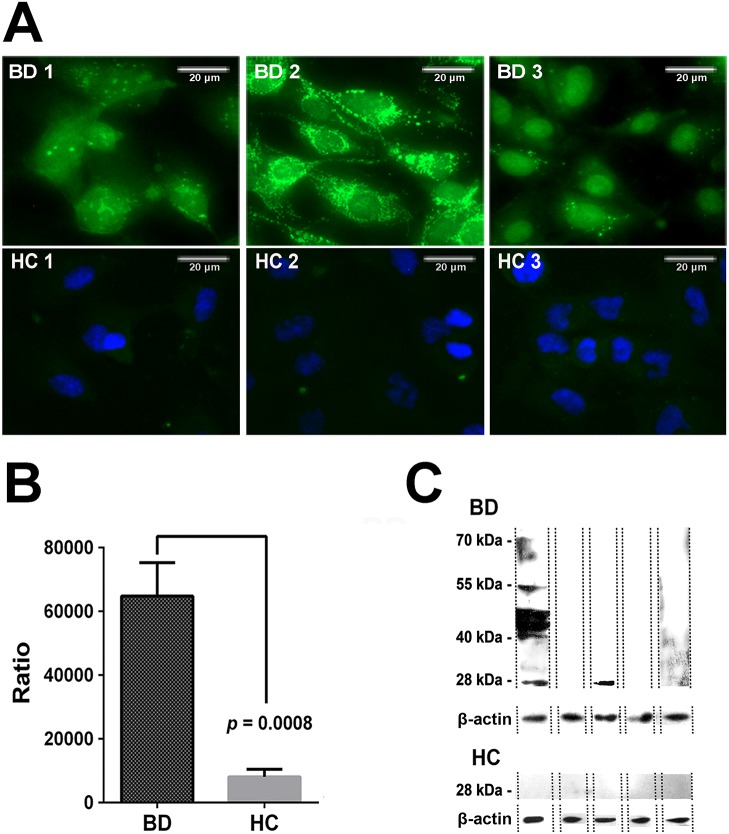
Indirect immunofluorescence assay and autoantigen detection. (A) HUVECs were subjected to an indirect immunofluorescence assay. The HUVECs had a strong positive reaction with sera from BD patients, indicating that autoantigens may be present in the sera. (B) The total cell fluorescence was obtained using Image J software. There were significant differences between the BD and HC groups (*P* = 0.0008). (C) Western blotting of HUVEC extracts with sera from the first batch of 5 BD patients revealed a positive band (≈ 28-kDa) in 2 patients (top row) but not in the HCs (bottom row). BD, Behçet’s disease; HC, healthy controls.

HUVECs were positively bound by the BD patient sera, which confirmed the presence of AECA in the sera (Fig 1A). The total difference in fluorescence between the BD and HC group was quantified by Image J software, and a significant difference was observed (*P* = 0.0008, Fig 1B). The existence of autoantigens in the cytoplasm of HUVECs was demonstrated by the presence of abundant fluorescence signals.

### Detection and identification of the autoantigens

**Fig 2:**
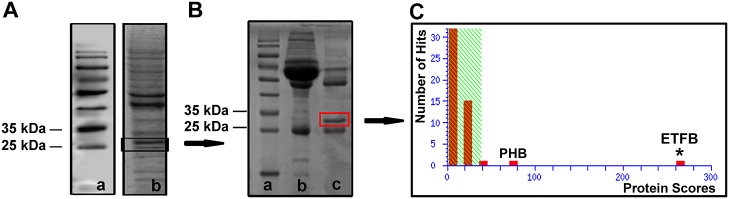
Identification of the positive 28-kDa band. (A) a: The molecular weight markers are shown on the left. b: Two positive bands at approximately 28 kDa were resolved and recovered. (B) Immunoprecipitation revealed an approximately 28-kDa protein band in the immunoprecipitate complex that reacted with the sera from BD patients. a: Protein marker. b: Supernatant. c: Immunoprecipitate complex. (C) The band at approximately 28 kDa was identified as the ETFB protein (NCBI accession number gi|4503609; www.ncbi.nlm.nih.gov). PHB: Prohibitin; ETFB: Electron transfer flavoprotein subunit beta

**Fig 3:**
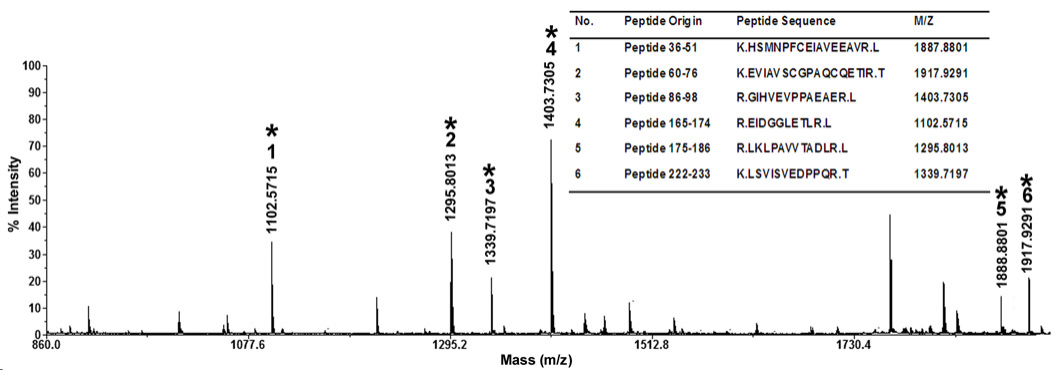
Unique peptides of the ETFB protein were identified by mass spectrometry. The peptide origin, amino acid sequences, and m/z are shown.

Western blotting was performed to detect the autoantigens associated with BD. IgG autoantibodies against the 28-kDa band were detected in 2 out of 5 BD patients but not in the 5 healthy controls (Fig 1C). Other bands were only observed in less one BD sample. So two different bands located at approximately 28-kDa were selected, separated and recovered for further immunoprecipitation (Fig 2A). The Western blotting result was verified in the immunoprecipitation. It was found that one approximately 28-kDa protein band reacted with the sera of BD patients (Fig 2B). Then, the autoantigen (≈28-kDa) was excised from a polyacrylamide gel and digested with trypsin. We performed LC-MALDI-TOF/TOF mass spectrometry and used the Mascot database scanning algorithm to identify the target protein. Two main proteins were identified in this band. One protein was found to share approximately 31% sequence similarity with human ETFB (NCBI accession number gi|4503609; Mascot score, 265; Fig 2C), and 6 unique peptides of ETFB were matched (Fig 3). Another protein was identified as prohibitin antigen with mascot score 90, which was reported in our previous study [12]. Our previous finding was reproduced in this independent experiment.

### ETFB is a new autoantigen in BD

**Fig 4:**
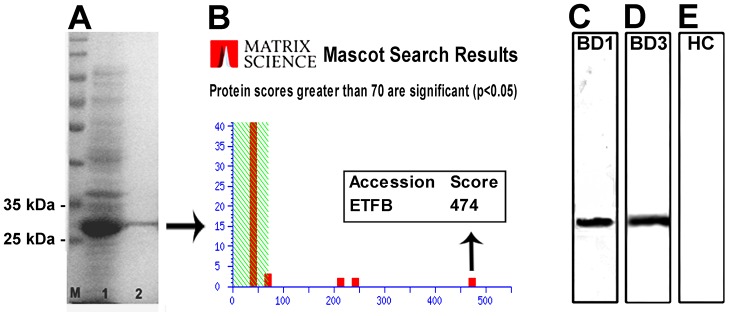
ETFB was confirmed as an autoantigen in BD. (A) The expression and purification of the ETFB protein. M: Marker; 1: *E*. *coli* BL21 containing a recombinant plasmid after the induction of expression with IPTG; 2: Purified recombinant ETFB protein. (B) Verification of the ETFB protein by mass spectrometry. The Mascot score was 474 (Matrix Sciences, London, UK; www.matrixscience.com). (C) Western blotting results for ETFB probed with serum from patient BD1 (1:500 dilution). (D) Western blotting results for ETFB probed with serum from patient BD3. (E) Healthy control.

In this experiment, recombinant human ETFB protein was expressed and purified in our lab, and it was then confirmed by MS (Fig 4A and 4B).

Western blotting analysis was performed on 2 μg of recombinant human ETFB protein with BD sera, and the patient sera positively recognized the recombinant ETFB, which supported the previous results obtained with native proteins from HUVECs Fig (4C–4E) and successfully confirmed that ETFB is a potential autoantigen in BD.

### ELISA results

**Fig 5:**
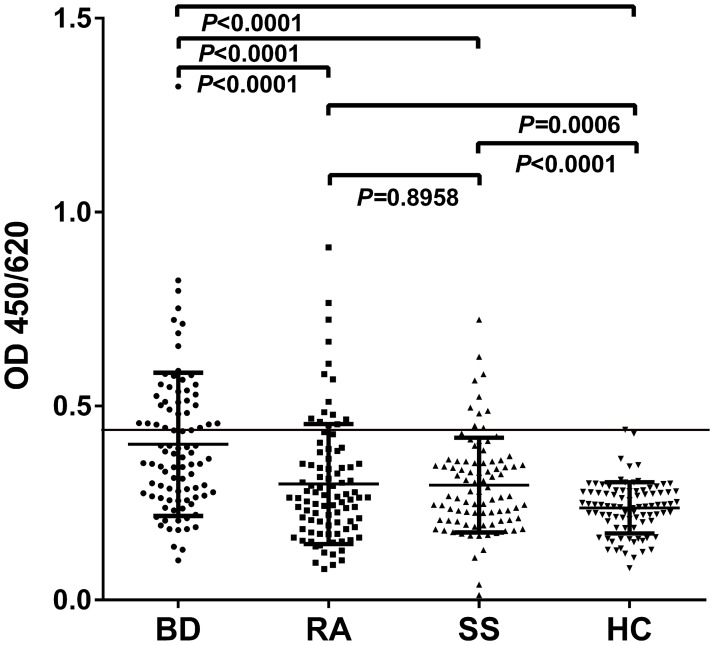
Reactivity of serum antibodies against ETFB. An ELISA was performed to detect the reactivity of the serum IgG antibodies against the human recombinant EFTB protein. The anti-ETFB antibodies were detected in 38 of 92 BD patients (41%), 16 of 92 RA patients (17%), 10 of 90 SS patients (11%), and 1 of 90 HC patients (1%). The reactivity of serum IgG to human recombinant ETFB in BD was significantly higher than in RA (*P*<0.0001), SS (*P*<0.0001) or HC (*P*<0.0001). The data are shown as the mean±SD. The threshold for defining a positive result was a higher value than that obtained for the healthy controls (mean + 3 SD), which was shown as horizontal line. BD, Behçet’s disease; HC, healthy controls; RA, rheumatoid arthritis; SS, Sjogren syndrome.

ELISA was performed to determine the prevalence of the anti-ETFB autoantibody in BD patient sera. We observed that 38 of 92 patients with BD (41%), 16 of 92 with RA (17%), 10 of 90 with SS (11%), and 1 of 90 HC (1%) were anti-ETFB positive, indicating that the anti-ETFB antibody had a significantly higher prevalence in BD than RA (*P*<0.0001), SS (*P*<0.0001) or HC (*P*<0.0001), which further confirmed that ETFB is an autoantigen in BD (Fig 5).

### Analysis of clinical characteristics

Among the 92 Han Chinese BD patients, 38 patients were anti-ETFB positive in the ELISA. Detailed clinical information was obtained from these patients, and their symptoms were observed (Table 1). By comparing the clinical information between the anti-ETFB antibody-positive and anti-ETFB antibody-negative groups, there were significantly more BD patients with gastrointestinal involvement in the anti-ETFB antibody-positive group than in the anti-ETFB antibody-negative group (*P*<0.05).

**Table 1:** Clinical characteristics of BD.

Characteristics	Patients with anti-ETFB IgG (n = 38)	Patients without anti-ETFB IgG (n = 54)	*P*
Age range	18–66	14–59	0.095
Sex, Male/Female	21/17	33/21	0.575
Recurrent oral ulcers **%**	95	98	1.000
Recurrent genital ulcers **%**	90	78	0.067
Anterior uveitis **%**	10	24	0.099
Nervous involvement **%**	18	15	0.645
Urinary involvement **%**	8	5	0.685
Joint involvement **%**	10	23	0.405
Lung involvement **%**	0	2	1.000
Vascular involvement %	26	17	0.260
Gastrointestinal involvement %	18	4	0.047

The clinical characteristics were analyzed by the chi-squared test, and age was analyzed using a t test with SPSS software (Version 17, Chicago, IL).

## Discussion

Electron transfer flavoprotein (ETF) was discovered to be capable of transferring the reducing equivalents derived from mammalian fatty acyl-CoA dehydrogenase to various electron acceptors [19, 20]. In mitochondria, the reducing equivalents from these dehydrogenases are transferred sequentially to ETF-ubiquinone oxidoreductase (ETF-QO). The ETF/ETF-QO system serves as a short electron transfer pathway to conduct electrons from primary flavoprotein dehydrogenases to the ubiquinone pool of the main respiratory chain [21, 22]. Defects in ETF or ETF-QO result in glutaric acidemia type II, a common fatal disease resulting from the inability to catabolize various fatty acyl-CoAs [23, 24]. Here, we first provided evidence that ETFB may be a candidate autoantigen in BD, thus extending our current knowledge of the ETFB protein. ETFB is a subunit of ETF [25]. However, it is unclear how ETFB acts as an autoantigen in BD.

The direct attack of normal host targets by autoantibodies is a common feature of many autoimmune diseases. Some of these autoantibodies are pathogenic, whereas others serve as biomarkers of organ involvement [26]. Autoantibody attack against ETFB may disrupt normal physiological functions in humans. Among the 92 BD patients included in this study, 38 patients showed a positive reaction to the recombinant human ETFB protein. By comparing clinical information between the anti-ETFB antibody-positive and anti-ETFB antibody-negative groups, there was a striking difference in gastrointestinal involvement between the anti-ETFB antibody-positive group and the antibody-negative group (*P*<0.05). Based on our current knowledge, the clinical manifestations (specific organ involvement) of many diseases is not consistent with the antigens’ expression (distribution) [27]. For example, a-enolase is also an AECA autoantigen in BD associated with intestinal syndrome [28]; however, it is widely expressed in many other cells.

The limitations of the present study should be acknowledged. More detailed clinical information must be analyzed in the next study. Overall, based on the results of this study, we suggest that ETFB may be a new autoantigen in BD. We will perform further investigations with additional patients enrolled from more clinical centers to evaluate and analyze anti-ETFB autoantibody induction in multiple autoimmune diseases.

## References

[1] Sakane T, Takeno M, Suzuki N, Inaba G. Behçet's disease. N Engl J Med. 2011;341: 1284–1291. 10.1056/NEJM199910213411707 10528040

[2] Verity DH, Marr JE, Ohno S, Wallace GR, Stanford MR. Behçet’s disease, the Silk Road and HLA-B51: historical and geographical perspectives. Tissue Antigens. 1999;54: 213–220. 10519357 10.1034/j.1399-0039.1999.540301.x

[3] Chambrun DMP, Wechsler B, Geri G, Cacoub P, Saadoun D. New insights into the pathogenesis of Behcet's disease. Autoimmun Rev. 2012;11: 687–698. 10.1016/j.autrev.2011.11.026 22197900

[4] Mor F, Weinberger A, Cohen IR. Identification of alpha-tropomyosin as a target self-antigen in Behçet's syndrome. Eur J Immunol. 2002;32: 356–365. 11807775 10.1002/1521-4141(200202)32:2<356::AID-IMMU356>3.0.CO;2-9

[5] Verity DH, Wallace GR, Vaughan RW, Stanford MR. Behçet’s disease: from Hippocrates to the third millennium. Br J Ophthal. 2003;87: 1175–1183. 12928293 10.1136/bjo.87.9.1175 PMC1771837

[6] Mendes D, Correia M, Barbedo M, Vaio T, Mota M, Gonçalves O, et al Behçet's disease-a contemporary review. J Autoimmun. 2009;32: 178–188. 10.1016/j.jaut.2009.02.011 19324519

[7] Dinc A, Takafuta T, Jiang D, Melikoglu M, Saruhan-Direskeneli G, Shapiro SS. Anti-endothelial cell antibodies in Behçet's disease. Clin Exp Rheumatol. 2003;21: S27–S30. 14727455

[8] Aydıntug AO, Tokgöz G, D'Cruz DP, Gürler A, Cervera R, Düzgün N, et al Antibodies to endothelial cells in patients with Behçet's disease. Clin Immunol Immunopathol. 1993;67: 157–162. 8519091 10.1006/clin.1993.1059

[9] Bax M, Huizinga TW, Toes RE. The pathogenic potential of autoreactive antibodies in rheumatoid arthritis. Semin Immunopathol. 2014;36: 313–325. 10.1007/s00281-014-0429-5 24763532

[10] Moutsopoulos HM. Sjögren's syndrome: A forty-year scientific journey. J Autoimmn. 2014;51: 1–9. 10.1016/j.jaut.2014.01.001 24485155

[11] Cho SB, Ahn KJ, Kim DH, Zheng Z, Cho S, Kang SW, et al Identification of HnRNP-A2/B1 as a target antigen of anti-endothelial cell IgA antibody in Behcet's disease. J Invest Dermatol. 2012;132: 601–608. 10.1038/jid.2011.397 22205302

[12] Xun Y, Chen P, Yan H, Yang W, Shi L, Chen G, et al Identification of prohibitin as an antigen in Behcet’s disease. Biochem Biophys Res Commun. 2014;451: 389–393. 10.1016/j.bbrc.2014.07.126 25091478

[13] Chen P, Shi L, Jiang Y, Ji Y, Yan H, Sun S, et al Identification of heat shock protein 27 as a novel autoantigen of Behçet’s disease. Biochem Biophys Res Commun. 2015; 456: 866–871. 10.1016/j.bbrc.2014.12.064 25529454

[14] Karasawa R, Yudoh K, Ozaki S, Kato T. Anti-endothelial cell antibodies (AECA) in patients with systemic vasculitis: our research using proteomics Expert Opin Biol Ther. 2011;11: 77–87. 10.1517/14712598.2011.540234 21133816

[15] International Study Group for Behçet's Disease. Criteria for diagnosis of Behçet's disease. Lancet. 1990;335: 1078–1080. 1970380

[16] Du H, Li C, Jin H, Chen G, Xun Y. Generation and evaluation of antibodies against human MGF E-peptide by reverse phase protein microarray and reverse competitive ELISA. Bioanalysis. 2013;5: 2269–2275. 10.4155/bio.13.195 24053242

[17] Deng C, Deng A, Sun S, Wang L, Wu J, Wu Y, et al The Periplasmic PDZ Domain-Containing Protein Prc Modulates Full Virulence, Envelops Stress Responses, and Directly Interacts with Dipeptidyl Peptidase of Xanthomonas oryzae pv. oryzae. Mol Plant Microbe Interact. 2014; 27: 101–112. 10.1094/MPMI-08-13-0234-R 24200074

[18] Du H, Chen G, Wang S, Wang S, Li S, Li C, et al Immunological screening and characterization of highly specific monoclonal antibodies against 20 kDa hGH. Bioanalysis. 2012;4: 2161–2168. 23013398 10.4155/bio.12.188

[19] Crane FL, Beinert H. On the mechanism of dehydrogenation of fatty acyl derivatives of coenzyme A. J Biol Chem. 1956;218: 701–716. 13295224

[20] Beinert H, Frisell WR. The functional identity of the electron-transferring flavoproteins of the fatty acyl coenzyme A and sarcosine dehydrogenase systems. J Biol Chem. 1962;237: 2988–2990. 13866632

[21] Ghisla S, Thorpe C. Acyl-CoA dehydrogenases. Eur J Biochem. 2004;271: 494–508. 14728676 10.1046/j.1432-1033.2003.03946.x

[22] Watmough NJ, Frerman FE. The electron transfer flavoprotein: ubiquinone oxidoreductases. Biochim Biophys Acta. 2010;1797: 1910–1916. 10.1016/j.bbabio.2010.10.007 20937244

[23] Loehr JP, Goodman SI, Frerman FE. Glutaric acidemia type II: heterogeneity of clinical and biochemical phenotypes. Pediatr Res. 1990;27: 311–315. 2320399 10.1203/00006450-199003000-00024

[24] Schiff M, Froissart R, Olsen RK, Acquaviva C, Vianey-Saban C. Electron transfer flavoprotein deficiency: functional and molecular aspects. Mol Genet Metab. 2006;88: 153–158. 16510302 10.1016/j.ymgme.2006.01.009

[25] Roberts DL, Frerman FE, Kim JP. Three-dimensional structure of human electron transfer flavoprotein to 2.1-Å resolution. Proc Nati Acad Sci USA. 1996;93: 14355–14360. 8962055 10.1073/pnas.93.25.14355 PMC26136

[26] LEE S, KAVANAUGH A. Autoimmunity, vasculitis, and autoantibodies. J Allergy Clin Immunol. 2006;117: S445–S450. 16455344 10.1016/j.jaci.2005.06.023

[27] Mak TW, Saunders ME. Primer to the immune response: New York: Academic Press; 2008.

[28] Shin SJ, Kim BC, Kim TI, Lee SK, Lee KH, Kim WH. Anti-alpha-enolase antibody as a serologic marker and its correlation with disease severity in intestinal Behçet’s disease. Dig Dis Sci. 2011;56: 812–818. 10.1007/s10620-010-1326-y 20632102

